# BIO101 in Sarcopenic Seniors at Risk of Mobility Disability: Results of a Double‐Blind Randomised Interventional Phase 2b Trial

**DOI:** 10.1002/jcsm.13750

**Published:** 2025-03-03

**Authors:** Roger A. Fielding, Michael M. Dao, Kevin Cannon, Moise Desvarieux, Sam S. Miller, Michael Paul Gimness, Donald M. Brandon, Dennis T. Villareal, Olivier Bruyere, Ivan Bautmans, Kyle Rickner, Robert Perry, Stephen B. Kritchevsky, Nicolas Musi, Joe M. Chehade, Judith L. Kirstein, Evelien Gielen, Paul Pickrell, Pierre Dilda, Rene Lafont, Carole Margalef, Yves Rolland, Susanna Del Signore, Jean Mariani, Samuel Agus, Cendrine Tourette, Waly Dioh, Rob van Maanen, Stanislas Veillet

**Affiliations:** ^1^ Nutrition, Exercise Physiology, and Sarcopenia Laboratory Jean Mayer USDA Human Nutrition Research Center on Aging at Tufts University Boston Massachusetts USA; ^2^ AMD Medical Group and National Institute of Clinical Research Inc. Garden Grove California USA; ^3^ PMG Research of Wilmington Wilmington North Carolina USA; ^4^ Department of Epidemiology, Mailman School of Public Health Columbia University New York New York USA; ^5^ METHODS Core, Centre de Recherche Epidémiologie et Statistique Paris Sorbonne Cité (CRESS), Institut National de la Santé et de la Recherche Médicale (INSERM) UMR 1153 Paris France; ^6^ SAM CLINICAL RESEARCH CENTER/Science Advancing Medicine San Antonio Texas USA; ^7^ Family Medical Specialists of Florida Plant City Florida USA; ^8^ California Research Foundation San Diego California USA; ^9^ Center for Translational Research on Inflammatory Diseases Michael E DeBakey Veterans Affairs (VA) Medical Center Houston Texas USA; ^10^ Research Unit in Public Health, Epidemiology and Health Economics University of Liège Liège Belgium; ^11^ Frailty & Resilience in Ageing Research Unit (FRIA), Vitality Research Group, and Gerontology Department Vrije Universiteit Brussel Brussels Belgium; ^12^ Department of Geriatric Physiotherapy SOMT University of Physiotherapy Amersfoort The Netherlands; ^13^ Geriatrics Department Universitair Ziekenhuis Brussel Brussels Belgium; ^14^ Tekton Research Yukon Oklahoma USA; ^15^ Panax Clinical Research Miami Lakes Florida USA; ^16^ Section on Gerontology and Geriatric Medicine, Department of Internal Medicine Wake Forest University School of Medicine Winston‐Salem North Carolina USA; ^17^ Department of Medicine Cedars‐Sinai Medical Center Los Angeles California USA; ^18^ Department of Medicine University of Florida College of Medicine Jacksonville Florida USA; ^19^ Velocity Clinical Research Banning California USA; ^20^ Department of Geriatric Medicine, UZ Leuven, & Department of Primary Care and Public Health, Division of Gerontology and Geriatrics KU Leuven Leuven Belgium; ^21^ Tekton Research Austin Texas USA; ^22^ BIOPHYTIS SA Sorbonne Université Paris France; ^23^ FSI, Paris‐Seine Biology Institute (BIOSIPE), CNRS Sorbonne Université Paris France; ^24^ IHU HealthAge, Centre Hospitalo‐Universitaire de Toulouse; CERPOP UMR 1295 University of Toulouse III Toulouse France; ^25^ Bluecompanion Ltd London UK; ^26^ Bluecompanion France Jambville France; ^27^ CNRS ‐ Institut de Biologie Paris Seine (UMR DEV2A) Sorbonne Université Paris France

**Keywords:** 20‐hydroxyecdysone, BIO101, clinical trial, gait speed, mobility disability, sarcopenia

## Abstract

**Background:**

Sarcopenia is a progressive muscle disorder that may lead to mobility disability. No pharmaceutical interventions are currently available, and treatment relies on physical exercise and nutrition. The aim of SARA‐INT was to investigate whether BIO101 (20‐hydroxyecdysone), an activator of the MAS receptor, is safe and improves muscle function and physical performance of community dwelling older sarcopenic patients.

**Methods:**

SARA‐INT was a randomised three‐arm interventional study (BIO101 175 mg bid /350 mg bid/placebo) with a planned 6‐month treatment (up to 9 months in 50 subjects). Eligibility criteria for sarcopenia were meeting FNIH criteria for sarcopenia and Short Physical Performance Battery (SPPB) score ≤ 8/12 in men and women aged ≥ 65 years. Primary endpoint was the change from baseline (CFB) in gait speed (GS) measured by 400‐m walking test (400MWT), secondary endpoints being CFB in other physical performance tests.

**Results:**

A total of 233 participants were randomised (mean age 75.5 ± 7.12; 54.3% female), of whom 232 and 156 were included in the full analysis set (FAS) and per‐protocol (PP) populations, respectively. Due to COVID‐19 pandemic, 55% of on‐site end‐of‐treatment efficacy assessments were lost, reducing the studies' power. In the primary analysis (mix of 6/9 months), BIO101 350 mg bid treatment after 6/9 months was associated with an improvement in the 400MWT of 0.07 m/s versus placebo in the FAS population (not significant) and of 0.09 m/s in the PP population (*p* = 0.008). BIO101 350 mg bid treatment effect on the 400MWT GS was also observed in pre‐defined subpopulations at higher risk of mobility disability (0.0474 m/s for slow walkers, 0.0521 m/s for obese and 0.0662 m/s for chair stand sub‐score ≤ 2 from SPPB in the FAS population), with a trend for a dose response. BIO101 showed a good safety profile at both doses (number of subjects with related treatment emergent adverse events (TEAEs) of 13 (16.0%), 10 (13.3%) and 10 (13.5%) in the placebo, 175 mg and 350 mg BIO101 groups, respectively).

**Conclusions:**

After 6 to 9 months of treatment, BIO101 350 mg bid showed strong trends consistent with a clinically relevant effect on the 400MWT GS, close to the minimal clinically important difference (MCID) in sarcopenia (0.1 m/s). This was also shown in predefined subpopulations at higher risk of mobility disability. BIO101 showed a good safety profile. Taken together, efficacy and safety data of this Phase 2 trial encourage us to pursue further development of BIO101 for the treatment of sarcopenia.

## 1 Introduction

### 1.1 Sarcopenia

Sarcopenia is a muscle disorder characterised by a progressive loss of muscle mass and function, usually beginning to develop by the fifth decade. Optimal care for people with sarcopenia is essential because this condition has high personal, social and economic burdens when untreated. In terms of human health, sarcopenia increases the risk of falls and fractures; impairs ability to perform activities of daily living; is associated with cardiac disease, respiratory diseases and cognitive impairment; leads to mobility disorders; and contributes to lowered quality of life, loss of independence and need for long‐term care placement and death. It is recognised as one of the five pillars of frailty [1].

In 2016, the Center for Disease Control and Prevention established an ICD‐10‐CM code for sarcopenia (ICD10‐CM diagnosis code M62.84), thereby providing its recognition as a clearly defined disease and for separate reporting and data collection. Depending on the cut‐offs employed, sarcopenia prevalence in 60–70‐year‐olds was reported as 5% to 13%, while the prevalence ranged from 11% to 50% in people >80 years. The number of people around the world aged ≥ 60 years was estimated at 600 million in the year 2000, a figure that is expected to rise to 1.2 billion by 2025 and 2 billion by 2050. Even with a conservative estimate of prevalence, sarcopenia affects > 50 million people today and will affect > 200 million in the next 40 years [2]. According to the World Health Organization (WHO) in 2009, the estimated direct healthcare cost attributable to sarcopenia in the United States in 2000 was USD 18.5 billion [3]. In 2014, the Foundation for the National Institute of Health (FNIH) developed a definition based on a meta‐analysis of 11 clinical studies and 26 725 participants (mean age: 75.2 ± 6.1 years in men and 78.6 ± 5.9 years in women) [4]. This definition considered cut‐off values for weakness of grip strength, <26 kg for men and <16 kg for women, and for low lean mass, appendicular lean mass adjusted for body mass index (ALM/BMI) < 0.789 for men and < 0.512 for women [4]. For the purpose of the reported interventional clinical trials, these cut‐offs seemed suitable to identify sarcopenia as they take into account BMI.

### 1.2 Current Status of Interventions for Sarcopenia

Several classes of medicines have been evaluated with different mechanisms of action, for example testosterone [5] and selective androgen receptor modulators (SARMS) [6, 7] and drugs that target the myostatin/activin pathway (activin receptor agonists, myostatin or activin inhibitors [8, 9], skeletal muscle fast troponin activators). While some of these drugs have been effective in improving muscle mass and/or strength, clinically relevant improvements in physical performance has not been shown [10]. As of today, no pharmacological treatment has been approved for sarcopenia with only exercise and nutritional interventions showing some efficacy [11, 12, 13].

### 1.3 Drug Candidate BIO101

BIO101 is a drug candidate containing 20‐hydroxyecdysone (20E) purified at ≥ 97% as the active pharmaceutical ingredient. BIO101 targets the Mas receptor (MasR), on the protective arm of the renin angiotensin system (RAS) [14], where natural ligand is angiotensin 1–7. In pharmacological studies performed in C2C12 myotubes, BIO101 demonstrated EC_50_ values for fusion index, number of nuclei per myotube and myotube diameter of 0.75, 0.55 and 0.34 μM, respectively [15]. In human myocytes, BIO101 at 1 μM induced a hypertrophy with an increase in fusion index, in myotube sectional area and in number of nuclei per myotube [15]. The dose of 350 mg bid was selected as the highest dose to be tested in the Phase 2 study based on the results of a Phase 1 study in adult (≥ 18 years) including older (≥ 65 years) healthy volunteers showing a good safety and pharmacokinetic profile without effect on blood pressure [16] or other identified adverse drug reactions [17]. The dose of 175 mg bid was selected as second dose, a dose that was anticipated to be safe and well tolerated. In the present Phase 2 randomised trial, we evaluated the safety and efficacy of BIO101 for treating age‐related sarcopenia including sarcopenic obesity and assessed its efficacy on gait speed (GS) and other physical performance measures in at‐risk older adults living in the community.

## 2 Material and Methods

### 2.1 Study Design and Procedure

**FIGURE 1:**
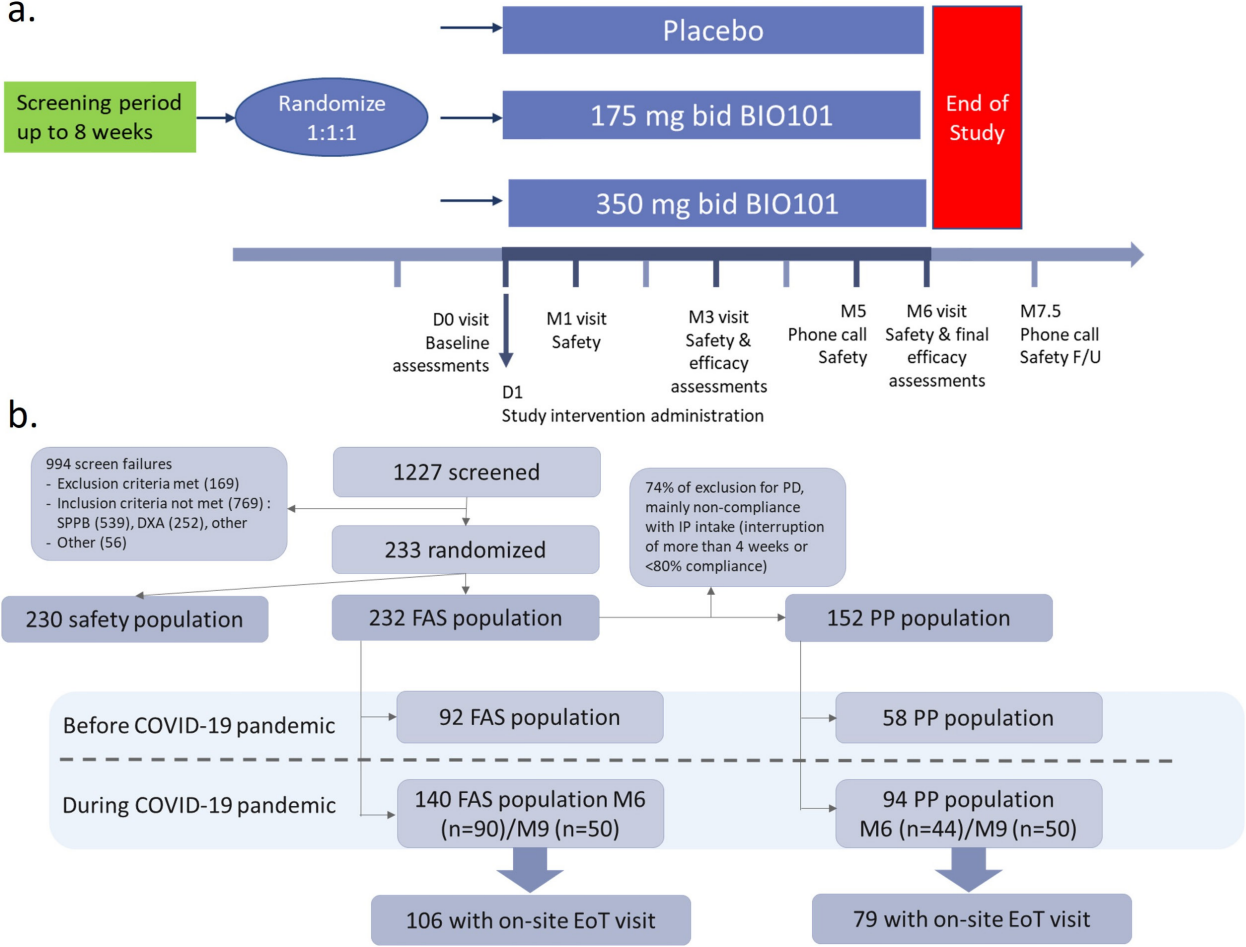
Study design and patient disposition. (a) Initial study design, including a screening period (up to 8 weeks, study intervention (26 weeks) and safety follow‐up of 6 weeks. (b) Patients disposition. EoT, end‐of‐treatment visit; FAS, full analysis set; PD, protocol deviation; PP, per protocol. After the COVID‐19 pandemic started, participants still active in the study have been proposed to increase their participation duration up to 9 months.

SARA‐INT was a randomised, double‐blind, placebo‐controlled study, with a planned treatment period of 26 weeks. Participants were randomly assigned (1:1:1) to receive orally BIO101 175 mg bid, BIO101 350 mg bid or placebo (Figure 1a) and were assessed regularly (Table S1). Owing to our concern for the safety of our older patients, clinic closures due to COVID‐19 pandemic compromised the on‐site visits of participants and an extension of treatment up to 39 weeks was proposed to participants who were participating in the treatment period on the date of 20 March 2020, aiming to obtain sufficient efficacy data.

Participants were randomised using an automated system that assigned participants to treatment arms with sex and center as stratification factors. All participants, investigators and sponsor representatives associated with the study were masked to the treatment allocation.

Site's institutional review boards/ethics committees approved the study protocol and amendments. The study was conducted in accordance with the International Council for Harmonization (ICH) guidelines for Good Clinical Practice and the Declaration of Helsinki. The study was registered in ClinicalTrials.gov (NCT 03452488) and EU Clinical trials register (EudraCT 2017‐003932‐35). All participants provided written informed consent before randomisation.

### 2.2 Participants

Participants were community‐dwelling individuals aged 65 and older suffering from sarcopenia, defined as having a Short Physical Performance Battery (SPPB) [18] score of 8 or below; low appendicular lean mass (ALM) assessed by dual‐energy X‐ray absorptiometry (DXA), following the cut‐off of the FNIH sarcopenia project [4]: ALM/BMI < 0.789 in males and 0.512 in females, or ALM < 19.75 kg in males and <15.02 kg in females, as measured by DXA scan and ability to complete the 400MWT in less than 15 min without sitting, stopping for more than 1 min, receiving help from another person or using a walker, and who reported a loss of physical function over the last 6 to 12 months. Main exclusion criteria were the concomitant use of anabolic drugs, erythropoietin or corticosteroids, diagnosis of major psychiatric disorders, severe arthritis, cancer requiring active treatment, lung disease requiring regular use of oxygen, severe cardiovascular disease, Parkinson's disease, renal disease requiring dialysis, active signs or symptoms of gallbladder/biliary disease. Participants were advised on proper nutrition and exercise and asked to avoid a sedentary lifestyle and encouraged to perform at least 30 min of physical activity (e.g., walking, gardening, light exercise) per day, at least 5 days per week. The compliance with this advice was not monitored.

### 2.3 Study Endpoints

The primary endpoint was the change from baseline (CFB) in GS in the 400MWT, with the primary analysis at month 6/9. This test was performed at each clinical trial site. The subjects walked 400 m on a 20‐m walking course (20 laps of 20 m) following a 2‐min warm‐up with standard encouragement from the trial team. Instructions were to walk at their usual pace.

The two key secondary endpoints were (1) CFB of patient‐reported quality of life (PF‐10 domain of the self‐administered SF36 questionnaire at Months 3, 6 and/or end of study [EoS]) and (2) CFB of handgrip strength (HGS) using a Jamar dynamometer. Grip strength was measured as the highest value of three assessments for both hands and recorded in kilograms (NIHR Southampton procedure) at baseline and Month 6 or EoS.

Other secondary and exploratory efficacy endpoints were SPPB (total score, sub‐scores [4‐m walking test, repeated chair stands and standing balance] and GS from the 4‐m walking test in meter per second; lean mass measured with DXA scans; distance walked during the 6‐min walk test, and other physical performance tests [Stair climb power test (time to climb a series of 10 stairs], knee extension [isometric knee extension torque measured by a Biodex System isokinetic dynamometer chair], data not shown) and questionnaires (Pepper Assessment Tool for Disability [PAT‐D], Sarcopenia Quality of Life [SarQoL] and Test SIO Disabilità Obesità Correlata [TSD‐OC] for obese patients) (Table S1).

### 2.4 Sample Size Calculation

Initially, the sample size was calculated for a comparison test at a 0.05 two‐sided significance level, a power of 80%, to detect a difference of 0.05 m/s between active groups and placebo on the CFB in the 400MWT GS at 6 months, with a standard deviation (SD) of 0.13 (estimated from the VIVE2 trial [19]. However, preliminary analysis from observational trial data (SARA‐OBS, with approximately 105 completers, submitted elsewhere), suggested that the study population with similar eligibility criteria deteriorated by 0.05 m/s on the 400MWT in 6 months, with a SD of the CFB of 0.20. This led to recalculation with an expected improvement in BIO101‐treated group of 0.05 m/s as CFB, a power of 80% to detect a substantial difference of 0.10 m/s (minimal clinically important difference—MCID) [20, 21] between active groups and placebo on CFB in 400MWT GS at 6 months; 64 subjects/group were needed, giving a sample size of 231 subjects with 20% provision for premature withdrawals or lost‐to‐follow up. Of note, the intermediate data review of the observational study used for the power calculation were used before database lock and proper data cleaning.

### 2.5 Statistical Analysis

A mixed‐effects model repeated measurement (MMRM) model with fixed factors of treatment, centres, baseline score and sex was used (in SAS v9.3) to estimate the CFB of the 400MWT GS at Month 6/9 between each active arm and the placebo group (after adjustment for multiplicity due to the two doses of active treatment by the Hochberg procedure). Imputation models were used for missing data using multiple imputation (MI) for participants without on‐site visit data due to COVID‐19 pandemic and its related restrictions and adjusted Bayesian imputation for non‐completers who failed to complete the 400MWT. Details are provided in supplemental information.

The key secondary endpoints (CFB in HGS and Physical Function Domain [PF‐10] sub‐score of the SF‐36) were analysed with the same strategy as the primary endpoint (i.e., using a MMRM to estimate the difference in CFB at Month 6/9 between each active group and placebo). Trial populations were safety population (randomised participants who had received at least one dose, analysed as treated), the full analysis set (FAS) population (all randomised participants who took at least one dose of BIO101 or placebo and not withdrawn within the first week after randomisation (analysed as randomised), and the per‐protocol (PP) population, consisting of FAS subjects who did not have a major protocol deviation related to noncompliance with study drug administration.

Predefined subgroup analyses were performed on participants with a low GS at baseline (GS ≤ 0.8 m/s in a 4‐m walk test from SPPB); sarcopenic obesity (defined as having body fat mass > 25% in men and > 35% in women); chair stand sub‐score ≤ 2 from SPPB.

Safety analyses and impact of COVID‐19 pandemic are described in the Supplemental information.

## 3 Results

### 3.1 Baseline Characteristics and Patients Disposition

A total of 1227 subjects were screened, and 999 were excluded as screen failure; 233 (19.0%) subjects were randomised (Figure 1b) in 12 clinical centres in United States and Belgium. Most common screen failure reasons were failing an inclusion criterion (769 subjects), mostly SPPB total score (*n* = 539 subjects, 70%) and DXA (*n* = 252 subjects, 33%) and meeting an exclusion criterion (169 subjects). The majority of randomised subjects were female (119; 51.3%). The mean (SD) age was 76.0 (6.9) years. The baseline characteristics were similar across treatment groups. The mean (SD) BMI was 28.4 (5.9); the mean 400MWT GS was 0.83 (0.22) m/s, and the mean PF‐10 score was 51.3 (25.5) (Table 1). There were no major differences between treatment groups at baseline in terms of medical history (data not shown). From the 233 randomised subjects, 203 (87.1%) completed the trial. The most common reasons for discontinuation from the study were AEs (11; 4.7%), withdrawn consent (8; 3.4%) and other reasons (4; 1.7%) (Figure 1b).

**TABLE 1:** Baseline characteristics of the full analysis set population.

	Placebo (*N* = 81)	175 mg BIO101 (*N* = 75)	350 mg BIO101 (*N* = 76)	Total (*N* = 232)
Age in years (SD)	75.5 (7.12)	76.2 (7.10)	76.3 (6.38)	76 (6.86)
Age group 65–75 (%)	41 (50.6)	35 (46.7)	35 (46.1)	111 (47.8)
Age group +75 (%)	40 (49.4)	40 (52.3)	41 (53.9)	121 (51.2)
% Male	45.7	50.7	50	48.7
% Female	54.3	49.3	50	51.3
Height in cm (SD)	162 (9.91)	164 (8.43)	163 (10.49)	163 (9.64)
Weight in kg (SD)	74 (16.89)	75 (18.45)	78 (23.20)	76 (19.61)
BMI in kg/m² (SD)	28 (5.26)	28 (5.76)	29.2 (6.66)	28.3 (5.91)
Gait speed from 400MWT, in m/s (SD)	0.847 (0.21)	0.81 (0.22)	0.824 (0.21)	0.827 (0.22)
Patients with sarcopenic obesity (%)	58 (71.6)	53 (70.7)	57 (75)	168 (72.4)
SPPB total score (SD)	6.5 (1.3)	6.5 (1.3)	6.4 (1.4)	6.5 (1.3)
PF‐10 from SF‐36 (SD)	53.1 (26.91)	50 (25.99)	50.5 (23.76)	51.3 (25.54)
CIRS (SD)	7.9 (4.46)	8.3 (4.59)	8.3 (3.76)	8.2 (4.28)
PAT‐D (SD)	1.81 (0.66)	1.77 (0.72)	1.84 (0.65)	1.81 (0.67)
SF‐MNA (SD)	12.8 (1.40)	12.8 (1.39)	12.9 (1.42)	12.8 (1.40)
Study drug exposure in days (SD) [a]	171.1 (74.4)	173.3 (79.3)	170 (69.3)	171.5 (74.3)
Compliance in % (SD) [b]	94.9 (17.0)	100.4 (14.5)	95.9 (15.4)	97 (15.8)

*Note:* Sarcopenic obesity was defined as percentage of body fat mass of > 25% in men and > 35% of total body weight in women. [a] Extent of exposure (days) = date of last dose − date of first dose + 1 based on the number of subjects in the safety population; [b] Compliance (%) = 100 × [(total number of capsules dispensed) − (total number of capsules returned)]/(total number of capsules planned to be taken per day × duration of study drug exposure in days). Percentages (%) were based on the number of subjects in the safety population.

Abbreviations: 400MWT, 400 m walking test; BMI, body mass index; CIRS, Cumulative Illness Rating Scale; PAT‐D, Pepper Assessment Tool for Disability; PF‐10, physical function sub‐score of SF‐36; SD, standard deviation; SF‐36, Short Form 36; SF‐MNA, Short Form ‐Mini Nutritional Assessment; SPPB, Short Physical Performance Battery.

### 3.2 Primary Endpoint 400MWT Gait Speed

After 6 months, observed 400MWT GS in the FAS population suggested a deterioration in the placebo group only, with the change from baseline being −0.023 (0.128) m/s. At M6, the observed difference between treatment arms and placebo was 0.037 and 0.086 m/s for the 175‐ and 350‐mg BIO101 groups, respectively. The observed difference between treatment arms and placebo was 0.045 and 0.066 m/s for 175 and 350 mg BIO101, respectively, at M6/M9. In the PP population, the observed difference between treatment arms and placebo was −0.019 and 0.102 m/s for 175 and 350 mg BIO101, respectively, at M6 and 0.019 and 0.078 m/s for 175 and 350 mg BIO101, respectively, at M6/M9 (Figure S1).

**FIGURE 2:**
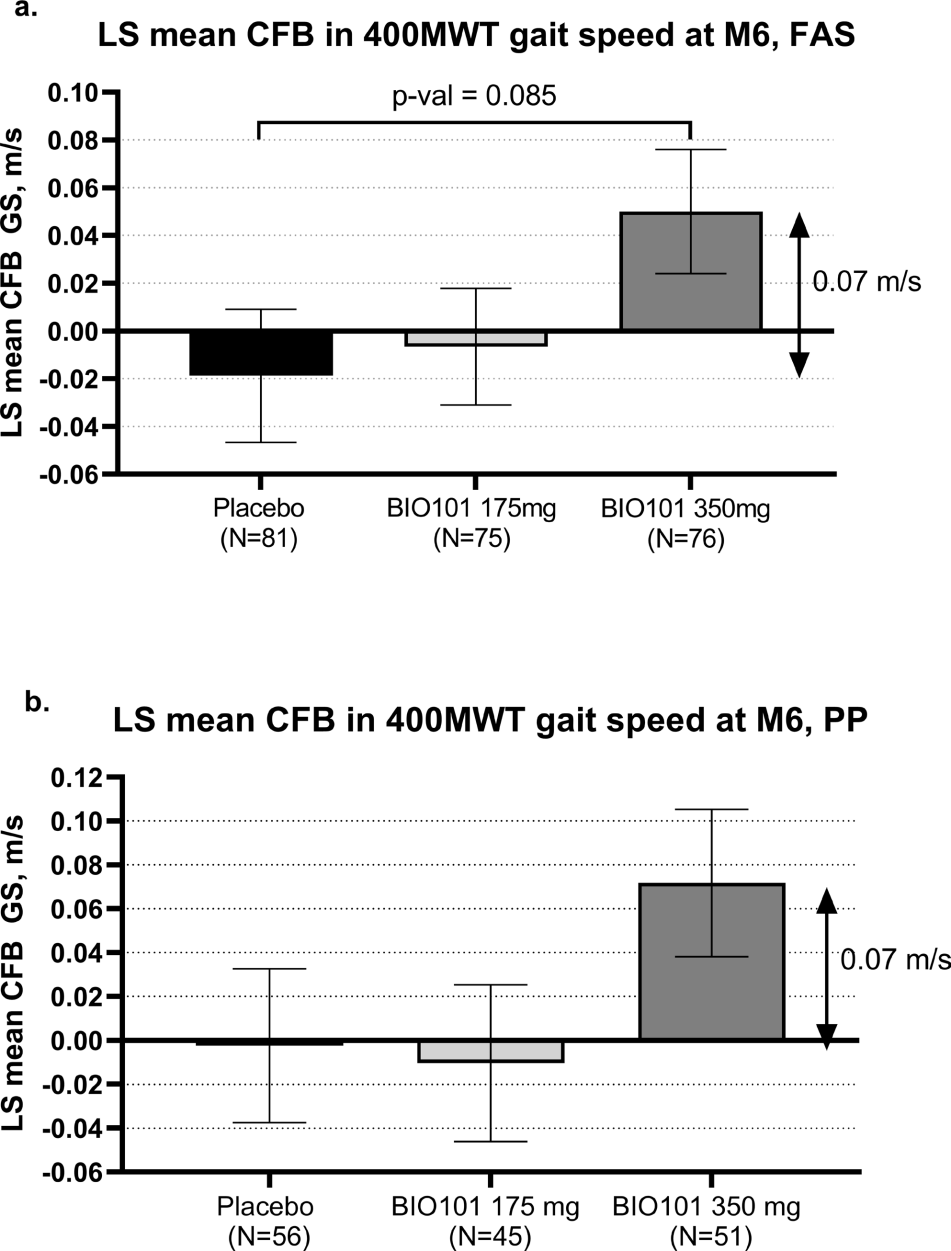
Change from baseline in 400MWT gait speed (SE), based on multiple imputation for subjects without on‐site visit data at Month 6 and adjusted Bayesian imputation for non‐completers at Month 6. (a) Full analysis set. (b) Per protocol population. LS, least square; SE, standard error.

The primary analysis included an adjusted Bayesian imputation for missing data, with a constraint that imputed values were < 0.44 m/s for the CFB of 400MWT GS. Missing data from patients who were not allowed to have on‐site visit due to COVID‐19‐related clinic closures were handled similarly. In the primary analysis of 400MWT GS in the FAS, the least square (LS) mean (SE) difference to placebo in change from baseline to Month 6/9 was 0.036 (0.031) m/s and 0.039 (0.030) m/s in the 175‐and 350‐mg BIO101 groups, respectively (*p* = 0.2437 and *p* = 0.2000, respectively). Missing data from patients who were not allowed to have on‐site visit due to COVID‐19‐related restriction were handled similarly. It was unforeseen and did not reflect adequately the walking ability of the subjects. New statistical analyses of CFB in 400MWT GS were conducted for subjects with baseline value, based on adjusted Bayesian imputation for non‐completers who failed to perform the test and MI for subjects without data of on‐site visit were applied: The LS mean (SE) difference to placebo in CFB to Month 6 was 0.012 (0.031) m/s and 0.069 (0.040) m/s in the 175‐ and 350‐mg BIO101 groups, respectively (*p* = 0.6920 and *p* = 0.0850, respectively) in the FAS population (Figure 2a). Similar results were obtained in the PP population (Figure 2b).

Predefined subgroups (4‐m GS ≤ 0.8 m/s, chair stand ≤ 2, obese) showed a potential treatment effect in the PP population at M6, see Table 2. In the subgroup with low GS, the LS mean (SE) difference versus placebo in CFB to Month 6 was −0.040 (0.056) m/s and 0.075 (0.049) m/s in the 175‐ and 350‐mg BIO101 groups, respectively (*p* = 0.4706 and *p* = 0.1395, respectively) (Table 2 and Figure S2). Overall, treatment was a significant factor in the MMRM analysis (*p* = 0.0154); however, centre and baseline were significant factors too (*p* = 0.0005 and *p* = 0.0108, respectively).

**TABLE 2:** Summary of subgroup analysis of the CFB at M6 of 400MWT gait speed in m/s, in FAS and PP populations.

Analysed subgroup	FAS			PP		
	Low gait speed (400MWT with a GS ≤ 0.8 m/s)					
Treatment arm	Placebo (*N* = 67)	175 mg BIO101 (*N* = 62)	350 mg BIO101 (*N* = 63)	Placebo (*N* = 47)	175 mg BIO101 (*N* = 37)	350 mg BIO101 (*N* = 42)
Number of subjects with Month 6 data	19	17	23	16	10	16
LS mean CFB M6 (SE), m/s	0.0220 (0.03341)	0.0326 (0.03626)	0.0694 (0.03215)	0.0279 (0.03415)	‐0.0125 (0.04532)	0.1024 (0.03677)
LS mean difference (vs placebo)(SE)		0.0105 (0.04709)	0.0474 (0.04417)		‐0.0404 (0.05550)	0.0745 (0.04940)
*p*‐value		0.8236	0.2878		0.4706	0.1395

Analysed subgroup	Sarcopenic obesity (percentage of body fat mass of >25% in men and > 35% in women)					
Treatment arm	Placebo (*N* = 58)	175 mg BIO101 (*N* = 53)	350 mg BIO101 (*N* = 57)	Placebo (*N* = 48)	175 mg BIO101 (*N* = 33)	350 mg BIO101 (*N* = 43)
Number of subjects with Month 6 data	21	19	26	18	11	19
LS mean CFB M6 (SE), m/s	0.0203 (0.02677)	0.0318 (0.02854)	0.0724 (0.02485)	0.0319 (0.02662)	−0.0359 (0.03601)	0.0888 (0.02753)
LS mean difference (vs placebo) (SE)		0.0115 (0.03722)	0.0521 (0.03505)		−0.0678 (0.04382)	0.0569 (0.03658)
*p*‐value		0.7592	0.1415		0.1278	0.1268

Analysed subgroup	Study subjects with a chair stand sub‐score of ≤ 2 of the SPPB at Screening					
Treatment arm	Placebo (*N* = 78)	175 mg BIO101 (*N* = 69)	350 mg BIO101 (*N* = 69)	Placebo (*N* = 53)	175 mg BIO101 (*N* = 41)	350 mg BIO101 (*N* = 45)
Number of subjects with Month 6 data	24	19	27	19	10	19
LS mean CFB M6 (SE), m/s	0.0018 (0.02903)	0.0523 (0.03325)	0.0680 (0.02939)	0.0139 (0.03104)	−0.0114 (0.04451)	0.1035 (0.03297)
LS mean difference (vs placebo)(SE)		0.0505 (0.04209)	0.0662 (0.03938)		−0.0254 (0.05318)	0.0895 (0.04429)
*p*‐value		0.2338	0.0966		0.6355	0.0492

*Note:* Analysis is based on mixed‐effect model for repeated measurements (MMRM) with treatment, visit, center, gender, and treatment * visit as fixed effects and baseline value as a covariate. The baseline value is defined as the last observation prior to or on the date of the first dose of study drug.

Abbreviations: CI, confidence interval; LS, least square; SE, standard error.

In the subgroup with chair stand sub‐score of ≤ 2 in SPPB, the LS mean (SE) difference compared with placebo in CFB to Month 6 was −0.025 (0.053) m/s and 0.090 (0.044) m/s in the 175‐ and 350‐mg BIO101 groups, respectively, statistically nominally significant for the latter (*p* = 0.6355 and *p* = 0.0492, respectively) (Table 2). In the subgroup with sarcopenic obesity, the LS mean (SE) difference versus placebo in CFB to Month 6 was −0.068 (0.044) m/s and 0.057 (0.037) m/s in the 175‐ and 350‐mg BIO101groups, respectively (*p* = 0.1278 and *p* = 0.1268, respectively) (Table 2). Overall, treatment was a significant factor in the MMRM analysis (*p* = 0.0037); however, centre and baseline were significant factors too (*p* = 0.0012 and *p* = 0.0417, respectively).

### 3.3 Secondary Endpoints

No significant differences between treatment arms and placebo for the secondary endpoints were observed: For the PF‐10 sub‐score from SF‐36 questionnaire, all groups increased their scores of 7.1 to 7.3; the LS mean (SE) difference to placebo in CFB to Month 6/9 was −0.2 (3.30) and −0.2 (3.25) in the 175‐ and 350‐mg BIO101 groups, respectively, which was not statistically significant (*p* = 0.9408 and *p* = 0.9485, respectively). In HGS at Month 6/Month 9, the CFB in the dominant hand was −0.709 (1.102), −1.601 (1.173) and 0.590 (1.067) in the placebo, 175‐ and 350‐mg BIO101 groups, respectively; the LS mean (SE) difference from placebo in CFB to Month 6/9 was −0.892 (1.381) and 1.298 (1.404) in the 175‐mg and 350‐mg BIO101 groups, respectively, which was not statistically significant (*p* = 0.5200 and *p* = 0.3577, respectively) (Table 3). Similar results were observed at Month 6 only (data not shown); none of these differences was statistically significant.

**TABLE 3:** Summary of statistical analyses of secondary endpoints with change from baseline at M6/M9 in the FAS population.

M6/9 in FAS population		Placebo (*N* = 81)	175 mg BIO101 (*N* = 75)	350 mg BIO101 (*N* = 76)
PF‐10 from SF‐36 mean (SD)	Number of subjects with M6/M9	33	33	37
	LS mean CFB M6/9 (SE)	7.3 (2.50)	7.1 (2.64)	7.1 (2.51)
	LS mean difference vs placebo		−0.2 (3.30)	−0.2 (3.25)
	*p*‐value		0.9408	0.9485
HGS dominant hand	Number of subjects with M6/M9	33	33	37
	LS mean CFB M6/9 (SE)	−0.709 (1.1024)	−1.601 (1.1731)	0.590 (1.0668)
	LS mean difference vs placebo (SE)		−0.892 (1.3807)	1.298 (1.4037)
	*p*‐value		0.5200	0.3577
ALM	Number of subjects with M6/M9	26	26	28
	LS mean CFB M6/9 (SE)	−0.276 (0.3176)	−0.283 (0.3307)	0.068 (0.2822)
	LS mean difference vs placebo (SE)		−0.007 (0.3859)	0.344 (0.4096)
	*p*‐value		0.9859	0.4040
ALM/BMI calculated (kg)	Number of subjects with M6/M9	26	26	28
	LS mean CFB M6/9 (SE)	−0.001 (0.0101)	−0.010 (0.0112)	0.007 (0.0097)
	LS mean difference vs Placebo (SE)		−0.009 (0.0131)	0.008 (0.0130)
	*p*‐value		0.4721	0.5554
400MWT test response	Number of responders with M6/M9	4	9	11
	Number of nonresponders with M6/M9	77	66	65
	Adjusted OR (95% CI) (vs placebo) [a]		2.57 (0.75–8.82)	3.15 (0.95–10.43)
	*p*‐value [a]		0.1321	0.0603
6MWD	Number of subjects with M6/M9	33	33	37
	LS mean CFB M6/9 (SE)	−9.879 (13.6981)	−7.166 (14.7976)	15.728 (13.4459)
	LS mean difference vs placebo (SE)		2.714 (18.2999)	25.607 (17.9817)
	p‐value		0.8824	0.1578
SPPB total score	Number of subjects with M6/M9	36	39	42
	LS mean CFB M6/9 (SE)	1.1 (0.35)	1.0 (0.35)	0.9 (0.33)
	LS mean difference vs placebo (SE)		−0.1 (0.47)	−0.2 (0.46)
	*p*‐value		0.8127	0.6663
SarQoL	Number of subjects with M6/M9	45	48	51
	LS mean CFB M6/9 (SE)	14.66 (2.017)	10.81 (2.074)	10.00 (1.979)
	LS mean difference vs placebo (SE)		−3.85 (2.559)	−4.67 (2.521)
	*p*‐value		0.1348	0.0666

*Note:* Analysis was based on mixed‐effect model for repeated measurements (MMRM) with treatment, visit, center, gender, and treatment * visit as fixed effects and baseline value as a covariate. The baseline value was defined as the last observation prior to or on the date of the first dose of study drug. Month 6/Month 9 data come from Month 6, but Month 9 value was used if Month 6 was missing. For the 400MWT test response, a responder was defined as an improvement (increase) of 0.1 m/s or more in 400MW gait speed test compared with baseline. A nonresponder was defined as a subject that was not a responder. Subjects with a missing value at the visit and/or missing baseline value were considered as nonresponders. The multiplicity issue due to the two doses of active treatment addressed using the Hochberg procedure. Percentages (%) were based on the number of subjects in the full analysis set. [a] Logistic regression using response (Y/N) as a response variable and treatment, gender and country as factors, and baseline score as covariate.

Abbreviations: 400MWT, 400 m walking test; 6MWD, 6‐min walking distance; ALM, appendicular lean mass; BMI, body mass index; CI, confidence interval; HGS, hand grip strength; LS, least square; OR, odds ratio; PF‐10, physical function sub‐score of SF‐36; SarQol, Sarcopenia Quality of Life questionnaire; SE, Standard Error; SF‐36, Short Form 36; SPPB, Short Physical Performance Battery.

**FIGURE 3:**
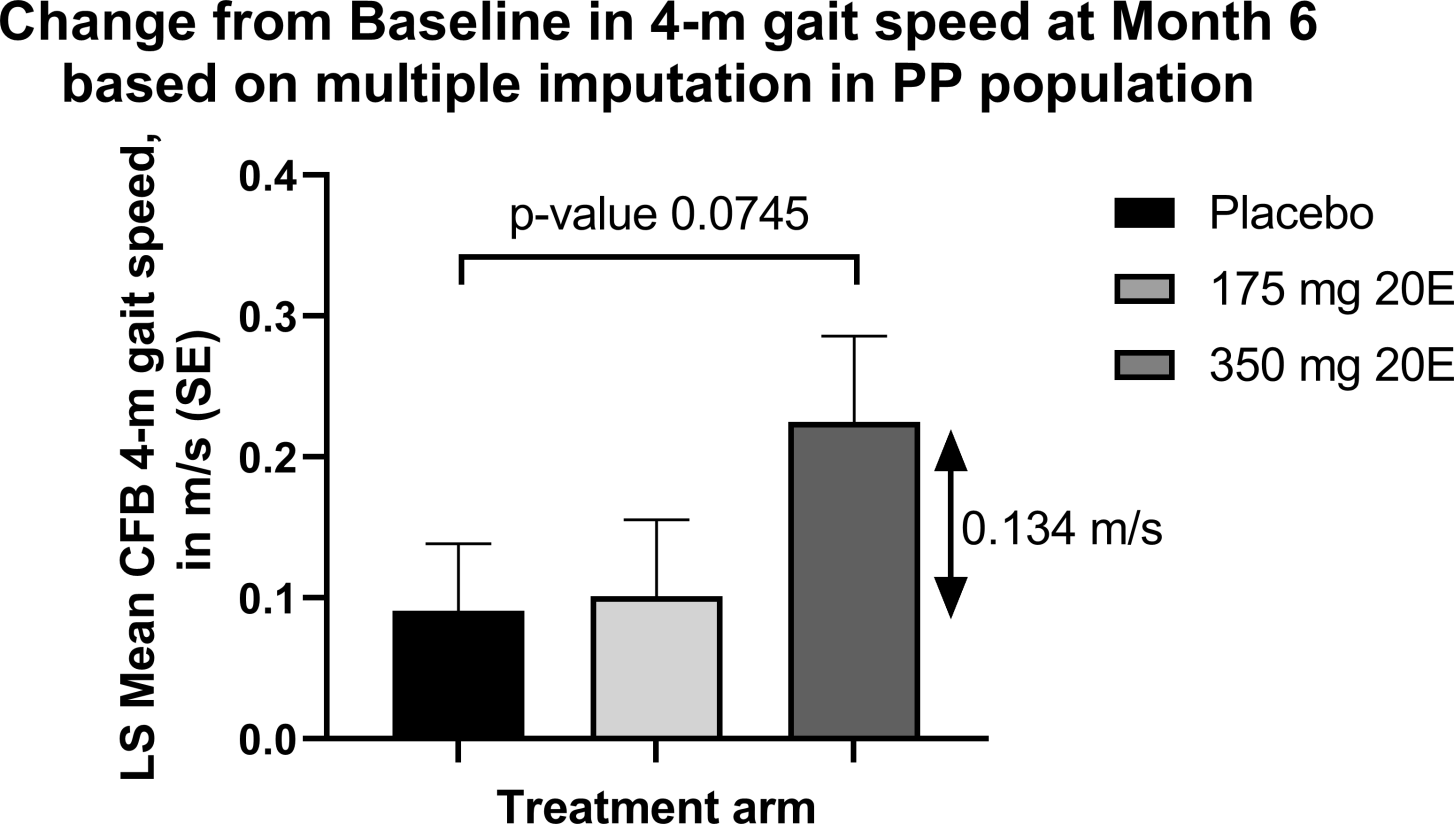
Change from baseline in 4‐m gait speed at Month 6 based on multiple imputation in the PP population. Missing data are imputed using multiple imputation. Analysis is based on mixed‐effect model for repeated measurements (MMRM) with treatment, visit, center, gender, and treatment * visit as fixed effects and baseline value as a covariate. LS, least square; SE, standard error.

Regarding other physical performance assessments, the LS mean (SE) difference to placebo at Month 6/9 in the distance walked during the 6‐min walking distance (6MWD) test was 2.714 m (18.300) and 25.607 m (17.982) in the 175‐ and 350‐mg BIO101 groups, respectively (*p* = 0.8824 and *p* = 0.1578, respectively). Other assessments did not demonstrate any difference between treatment arms and are summarised in Table 3. The LS mean (SE) difference to placebo at Month 6 in the GS from the 4‐m walk test from SPPB in the PP population was 0.011 (0.067) and 0.134 (0.074) m/s in the 175‐ and 350‐mg BIO101groups, respectively (*p* = 0.8747 and *p* = 0.0745, respectively) (Figure 3).

### 3.4 Safety Data

Overall, the number of subjects with AEs was 52 (64.2%), 51 (68.0%) and 44 (59.5%) in the placebo, 175‐ and 350‐mg BIO101 groups, respectively (Table 4). Of those, there were two treatment‐related serious adverse events (SAEs) in one subject in the placebo group; no subjects died during the treatment period; however, two subjects died outside the treatment period (one each with brain tumour and SARS CoV‐2 infection). Overall, there were eight (9.9%), 12 (16.0%) and eight (10.8%) subjects with treatment‐emergent adverse events (TEAE) that led to discontinuation from the trial in the placebo, 175‐ and 350‐mg BIO101 groups, respectively. The TEAEs, treatment‐related TEAEs, SAEs and AESI are summarised in Table 4. Of note, no subjects experienced any treatment‐related AESI. A summary of related TEAEs by SOC and preferred term is provided in Table S2. Overall, the proportion of subjects with related TEAEs (in the opinion of the investigator at site) was 13 (16.0%), 10 (13.3%) and 10 (13.5%) in the placebo, 175‐ and 350‐mg BIO101 groups, respectively. Most common were TEAEs of the System Organ Class (SOC) gastrointestinal disorders, 3 (3.7%), 4 (5.3%) and 5 (6.8%) subjects in the placebo, 175‐mg and 350‐mg BIO101 groups, respectively, followed by musculoskeletal and connective tissue disorders in two (2.5%), four (5.3%) and two (2.7%) subjects, respectively (Table 4).

**TABLE 4:** Summary of adverse events in the safety population.

Number of AEs/subjects with AEs	Placebo (*N* = 81)		175 mg BIO101 (*N* = 75)		350 mg BIO101 (*N* = 74)		Overall (*N* = 230)	
	AEs	*n* (%)	AEs	*n* (%)	AEs	*n* (%)	AEs	*n* (%)
Subjects with any TEAE	107	48 (59.3)	101	45 (60.0)	70	38 (51.4)	278	131 (57.0)
Subjects with any treatment‐related TEAE	24	13 (16.0)	15	10 (13.3)	16	10 (13.5)	55	33 (14.3)
Subjects with any treatment‐Rrelated serious TEAE	2	1 (1.2)	0	0	0	0	2	1 (0.4)
Number of TEAEs with maximum severity								
Mild	58	27 (33.3)	52	23 (30.7)	40	24 (32.4)	138	74 (32.2)
Moderate	27	12 (14.8)	21	9 (12.0)	19	11 (14.9)	65	32 (13.9)
Severe	13	9 (11.1)	15	13 (17.3)	4	3 (4.1)	32	25 (10.9)
Subjects with any SAEs	15	10 (12.3)	14	10 (13.3)	4	4 (5.4)	33	24 (10.4)
Subjects with any serious TEAE	13	9 (11.1)	14	10 (13.3)	2	2 (2.7)	29	21 (9.1)
Subjects with any treatment‐emergent adverse event of special interest (AESI)	12	7 (8.6)	11	8 (10.7)	11	8 (10.8)	34	23 (10.0)
Subjects with any serious AESI	2	1 (1.2)	1	1 (1.3)	0	0	3	2 (0.9)
Subjects with any TEAE leading to treatment discontinuation		8 (9.9)		12 (16.0)		8 (10.8)		28 (12.2)

*Note*: *n* = number of subjects in the specified category; *N* = group number. Percentage (%) based on the number of subjects in the safety population. TEAE were defined as any event that started on or after the first dose date of study drug up to the last dose date + 6 weeks (date of first randomized study medication intake ≤ AE onset date ≤ last dose date + 6 weeks).

### 3.5 Impact of COVID‐19‐Related Restrictions on Study Conduct

On‐site evaluations of participants (55%) from 16 March 2020 were lost. Sensitivity analyses for end‐of‐study before or after 16 March 2020 were performed and did not highlight any significant impact on evolution of the participants nor effect of BIO101 treatment, in both the FAS and PP population.

## 4 Discussion and Conclusions

### 4.1 Results

Overall, the clinical trial results were consistent in suggesting a dose‐related improvement in the physical performance assessments (400MWT, 6MWD, 4‐m GS from SPPB, HGS), despite the lack of statistical significance of most of the tests. The 400MWT is a well‐established assessment for sarcopenia, and GS from 400MWT has also been shown to correlate with mortality [22]. This distance corresponds to a quarter mile, representing the minimal distance to walk for a senior living autonomously in the community, used in self‐reported measures of mobility disability. Inability to complete this test in 15 min is accepted as an early clinical manifestation of the disability cascade [23, 24]. Improving the GS may reduce or delay the disability cascade. Of note, the deterioration of the GS in the placebo group is very similar to the data obtained from an observational study (−0.027 [0.171] m/s, published elsewhere). Trends observed in muscle strength might be promising as well, as HGS was also associated with disability [25], falls, self‐reported mobility limitation, hip fractures and mortality [26]. Limitations in the analysis of this endpoint relate to the high variability observed. Of note, no eligibility criterion was applied to muscle strength for this study. The relatively short duration of treatment (6 to 9 months) may also explain the lack of amplitude. Taken globally, and despite the unavailability of half of the end of treatment assessments due to COVID‐19, these data suggest a potential effect of BIO101 350 mg bid.

Due to the interference of pandemic on the clinical operation flow and daily activities of older patients, it is difficult to consider the adequacy of a 6‐month treatment versus a longer duration. The possibility to extend the active treatment beyond 6‐month should be considered when discussing the design of Phase 3 confirmatory studies in the target indication (age‐related sarcopenia).

An additional consideration for a Phase 3 study is the selection of a sensitive secondary endpoint that reflects the patient's view about a potential improvement.

Interestingly, subgroup analysis on the primary endpoint selecting the most frail subjects (chair stand ≤ 2, 4‐m GS < 0.8 m/s, sarcopenic obesity) suggests that BIO101 could be effective in the most frail population of sarcopenic patients.

Of note, no impact of BIO101 on lean mass was observed. The value of lean mass as measured with DXA has been debated since the beginning of the SARA‐INT study, with its low correlation with functional decline and poor health outcomes [27, 28, 29]. A proposal endorsed by leading experts in the field and incorporated in today's disease definition no longer features reduced muscle mass as diagnostic criterion [27]. Instead, the D3 creatine dilution assay, a more recent method for a muscle mass estimation, appears to be better associated with physical performance and muscle strength [26, 30, 31, 32], and thus may represent a more relevant measure of muscle mass for the next steps of the clinical development of BIO101.

To our knowledge, there is no report of efficacy on physical performance from clinical trials targeting sarcopenia using a pharmaceutical alternative [10]. The few clinical trials targeting sarcopenic population failed to show an effect on muscle strength and physical performance (Selective Androgen Receptor Modulator [SARM] [7]; Angiotensin Converting Enzyme Inhibitor [Perindopril] [33]; Bimagrumab [monoclonal antibody blocking activin receptor type IIA and IIB [9]]). Only Vitamin D administered with proteins seemed to be beneficial for physical performance [34]. Targeting a broader population, the ENRGISE study compared losartan and fish oil treatment versus placebo in older population with self‐reported mobility limitation, low GS and low‐grade chronic inflammation [35]. Neither losartan nor fish oil treatments over 12 months had an effect on 400MWT GS on this trial population, which was not selected based on any sarcopenia criteria. Only a nominally significant improvement in muscle power was observed in the highest dose enobosarm group compared with placebo in healthy elderly volunteers [6].

On the other hand, the LIFE study and its pilot study LIFE‐P proposed an intervention based on physical exercise targeting older adults at risk of mobility disability selected based on their SPPB total score only, without any other eligibility criterion related to sarcopenia. In the LIFE‐P study, participants with a SPPB total score of nine and below following an intervention based on physical exercise showed a difference in their 400MWT GS compared with the health education control arm of 0.03 m/s at 6 months and 12 months after randomisation [36]. The SPRINTT trial (Sarcopenia & Physical fRailty IN older people: multi‐component Treatment strategies) targeted physically frail and sarcopenic older adults. The data suggested a significant difference between multidomain intervention of lifestyle education for participants having a SPPB score of 3 to 7 at baseline (odds ratio of persistent mobility disability 0.79 [95% CI 0.62 to 1.01], *p*‐value of 0.06). This was consistent with the subgroup analysis in the SARA‐INT trial that highlighted the potential beneficial effect of an intervention on the most severe sarcopenic subgroups. In the subgroup with SPPB 3–7 in SPRINTT, the HGS showed a statistically significant difference between intervention arms only in females 24 months after randomisation (effect size [95% CI] of 0.9 [0.1 to 1.6], *p*‐value of 0.028), whereas no difference was detected in males [37]. In the SARA‐INT trial, no difference was detected between sexes, and no statistically significant difference was detected between 350 mg bid and placebo even if numerically similar to the SPRINTT population, suggesting a lack of power to detect an effect of BIO101 on muscle strength in this trial.

### 4.2 Safety

Overall, BIO101 was well tolerated and most TEAEs were mild, without any noticeable difference in TEAEs, related TEAEs or SAEs between treatment groups. BIO101 at the two doses showed a very good safety profile up to 9 months of administration, supporting longer exposure to the candidate drug in further steps of its clinical development.

### 4.3 Clinical Development

Taken globally and despite the unavailability of 55% of the endoftreatment assessments due to COVID‐19, although this study did not meet its primary outcome, it fell just short of the MCID in the PPl analysis, and thus, these data suggest a potential effect of BIO101 350 mg bid on physical performance of sarcopenic individuals. Interestingly, subgroup analysis on the primary endpoint selecting the most frail subjects (slow walkers with 4‐m GS ≤ 0.8, chair stand sub‐score from SPPB ≤ 2) suggested that BIO101 could be of particular interest in the most frail population of sarcopenic patients. This is consistent with the latest definitions of sarcopenia [27, 38, 39] and their overlap with physical frailty [1]. The results obtained encourage us to pursue the clinical development of BIO101 in sarcopenia.

### 4.4 Limitations

A limitation of the study lies on the evolution of the cutoff applied to define the target population. We followed the definitions focusing on lean mass and physical function (FNIH [4], EWSOP [40]), with restriction to a more vulnerable population using a lower SPPB cutoff (SPPB score ≤ 8). This was recently recommended by other consortia [38, 39]. The evolution of the cutoff applied might be taken into consideration when compared with other interventional studies.

Compliance with nutritional and exercise advice was not monitored, and exercise advice given may not have been feasible during the Covid‐19 containment, which may have impacted variability; nevertheless, the placebo‐controlled design ensured that this did not bias the results.

### 4.5 Impact COVID‐19

COVID‐19 restrictions had a critical impact on clinical assessments for 55% of randomised patients (hold of on‐site visits started on 16 March 2020). It was unforeseen and the primary analysis using adjusted Bayesian imputation for missing data did not reflect adequately the walking ability of the subjects. Also, the unforeseen severity and duration of the restrictions leading to a very different social context on the one hand and the difference of treatment duration M6/M9 on the other hand led to more variability. This led to an updated strategy for the final analysis and to the conduct of some post hoc analyses, including using a different imputation model based on the reason for missing data (MI when no on‐site visit due to COVID‐19/adjusted Bayesian imputation for non‐completer during on‐site visits). This loss greatly reduced the study's power and impacted the ability to detect the hypothesised treatment effect. Besides the loss of efficacy evaluation, sensitivity analyses performed on the impact of COVID‐19 (before/during COVID‐19 outbreak) did not yield any impact of COVID‐19 or its related restriction on the physical performance of this population. This may be explained by the low number of observations, the duration of follow‐up but also the high variability of local, US state and national recommendations in 2020.

## Conflicts of Interest

R.A.F. is partially supported by the US Department of Agriculture (USDA), under agreement No. 58‐8050‐9‐004, and by the NIH Boston Claude D. Pepper Center (OAIC; 1P30AG031679). Any opinions, findings, conclusions, or recommendations expressed in this publication are those of the authors and do not necessarily reflect the view of the USDA. R.A.F. reports grant support from Lonza, Biophytis, National Institutes of Health and USDA; scientific advisory board membership for Biophytis, Amazentis, Inside Tracker, Rejuventate Biomed and Aging in Motion; and consultancies for Embion, Biophytis, Amazentis, Pfizer, Nestle and Rejuvenate Biomed. Y.R. reports support from CHU Toulouse, University Paul Sabatier and INSERM CERPOP1295 (employee), to be a shareholder of SARQOL SPRL, a spin‐off of the University of Liege; consultancy fees from Longeveron, to be a Scientific Advisory Board member to Biophytis; and have received honoraria for lectures for Pfizer. OB reports to be stakeholder of SARQOL SRL, a spin‐off of the University of Liege in charge of the interest of the SarQoL and reports consulting or lecture fees (in the last 5 years) from Amgen, Aptissen, Biophytis, IBSA, Mylan, Novartis, Nutricia, Orifarm, Sanofi, UCB and Viatris. D.T.V. is on the scientific advisory board for Biophytis SA. E.G. reports consulting or lecture fees (in the last 5 years) from Amgen, Nutricia, Orifarm and UCB. I.B. reports to be stakeholder of SARQOL SRL, a spin‐off of the University of Liege in charge of the interest of the SarQoL. M.M.D., M.D., D.B., S.B.K., N.M., J.M.C., K.C., D.M.B., M.P.G., P.P., S.S.M., K.R., R.P. and J.K. declare that they have no competing interests. J.M. is the President of the Scientific Advisory Board of Biophytis SA. P.D., R.L., C.T., W.D., R.V.M. and S.V. are employees of Biophytis SA. C.M., S.D.S. and S.A. are former employees of Biophytis SA.

## Supporting information


**Data S1** Supplementary Information.

**Figure S1** Observed change from baseline at Month 6 of the 400MWT gait speed in meter per second (SEM) of the FAS population. Numbers indicate the number of observations at each timepoint and treatment arm.

**Figure S2:** Change from baseline in 400MWT gait speed (SE) at Month 6 in predefined subgroups: 400MWT gait speed ≤ 0.8 m/s at baseline (a), chair stand sub‐score ≤ 2 of SPPB (b) and sarcopenic obesity (c). Data are presented in FAS and PP populations.

**Table S1:** Schedule of activities. 400MWT, 400‐m walking test; AE, adverse event; CIRS, Cumulative Illness Rating Scale; DXA, dual‐energy X‐ray absorptiometry; EoS, end of study; ICF, Informed Consent Form; SarQoL: Sarcopenia Quality of Life questionnaire; SPPB, Short Physical Performance Battery; SF‐MNA, Short Form‐Mini Nutritional Assessment; SF36, Short Form 36; TSD‐OC, Test SIO Disabilità Obesità Correlata; PAT‐D, Pepper Assessment Tool for Disability.

**Table S2:** Summary of treatment‐emergent treatment related adverse events by system organ class and preferred term in the safety population. *N* = group number. (a) If a subject experienced more than one event within the same system organ class and preferred term, only one occurrence was included at each level of system organ class or preferred term. Totals for the number of subjects at system organ class level were not necessarily the sum of those at the preferred term levels because a subject could report two or more different adverse events within the higher‐level category. (b) The total number of events of the type specified. Subjects could be represented more than once. For ‘any treatment‐related TEAE’, it represents the total number of treatment‐related TEAEs. TEAE was defined as any event that starts on or after the first dose date of study drug up to the last dose date + 6 weeks (date of first randomised study medication intake ≤ AE onset date ≤ last dose date + 6 weeks). Adverse event was defined as related if causality was either definitely related, probably related or potentially related. Percentages (%) were based on number of subjects in the safety population.
