# Supplementary material for: BIO101 in Sarcopenic Seniors at Risk of Mobility Disability: Results of a Double‐Blind Randomised Interventional Phase 2b Trial
Source: J Cachexia Sarcopenia Muscle. 2025 Mar 3;16(2):e13750. doi: 10.1002/jcsm.13750 (PMC11873539; doi:10.1002/jcsm.13750)
Supplement: Supplementary file 1 — Data S1 Supplementary Information. [file JCSM-16-e13750-s001.docx]

**Supplemental information**

**Supplemental Material & methods**

**Study drug**

20-hydroxyecdysone (20E) was purified at ≥ 97% (Patheon, Germany) from a *Cyanotis* spp. extract (Nutragreen, Shanghai, China). Capsules containing 175 mg of BIO101 (20E) were produced at Amatsi, Saint-Gely (France). The matching placebo capsules contained the same excipients. Participants received in a blinded manner two capsules twice a day: 2 placebo, or 1 placebo and 1 BIO101 175 mg or 2 BIO101 175 mg, taken approximately twelve hours apart.

**Statistical analysis**

Imputation models were used for missing data using multiple imputation (MI) for participants without on-site visit data due to COVID-19 pandemic and its related restrictions and adjusted Bayesian imputation for non-completers who failed to complete the 400MWT (according to the method published by Chen et al. ^1^).

Missing data from patients who were not allowed to have on-site visit due to COVID‑19-related restriction were first handled similarly as patients who could not complete the test. It was unforeseen and did not reflect adequately the walking ability of the subjects. New statistical analyses of CFB in 400MWT GS were conducted for subjects with baseline value, based on Adjusted Bayesian Imputation for Non-Completers who failed to perform the test and MI for subjects without data of on-site visit were applied for M6 data.

Impact of COVID-19 pandemic

Impact of COVID-19 pandemic was assessed using sensitivity analyses comprising a COVID-19 outbreak classification: EoS before 16MAR2020 (before) and EoS after 16MAR (during pandemic). The last contact date was used if no EOS date were available (LOCF approach). COVID-19 Outbreak Classification was used for analysis of primary endpoint and key secondary endpoints in the FAS and PP population and AEs in the Safety Population.

**Safety**

Safety assessments included Adverse Events (AE) and Serious AE (SAE) coded in MedDRA (version 21.0), ECG (screening and baseline), vital signs (screening, baselines and EOS), biochemistry, hematology, coagulation, and urinalysis laboratory test results (at all on-site visits). Gallbladder ultrasound was performed at baseline to exclude previous or active hepatobiliary diseases (e.g. cholestasis/biliary tract obstruction, cholelithiasis, cholecystitis, etc.) and was repeated at the end of treatment to assess hepatobiliary abnormalities.

Orthostatic hypotension and falls/injurious falls were considered AE of Special Interest (AESI). Nutritional status was evaluated using the Short-Form-Mini Nutritional Assessment (SF-MNA)^2^. An independent Data Safety Monitoring Board reviewed quarterly the safety data of all participants in a blinded manner.

**Supplemental References**

1. Chen H, Ambrosius WT, Murphy TE, Fielding R, Pahor M, Santanasto AJ *et al.* Imputation of Gait Speed for Noncompleters in the 400-Meter Walk: Application to the Lifestyle Interventions for Elders Study. *Journal of the American Geriatrics Society* 2017;**65**:2566–2571.

2. Huhmann MB, Perez V, Alexander DD, Thomas DR. A self-completed nutrition screening tool for community-dwelling older adults with high reliability: a comparison study. *J Nutr Health Aging* 2013;**17**:339–344.

**Supplemental Figures legends**

**Figure S1:** Observed change from baseline at Month 6 of the 400MWT gait speed in m/s(SEM) of the FAS population. Numbers indicate the number of observations at each timepoint and treatment arm.

**Figure S2:** Change from baseline in 400MWT gait speed (SE) at month 6 in predefined subgroups: 400MWT gait speed ≤0.8m/s at baseline (a), chair stand sub-score ≤2 of SPPB (b), sarcopenic obesity (c). data are presented in FAS and PP populations.

**Supplemental Tables legends**

**Table S1**: Schedule of activities.

EoS: End of Study; ICF Informed Consent Form; SPPB Short Physical Performance Battery; DXA Dual energy X-ray Absorptiometry; 400MWT: 400-meter walking test; CIRS: Cumulative Illness Rating Scale; SF-MNA: Short Form -Mini Nutritional Assessment ; SF36: short form 36; SarQoL: Sarcopenia Quality of Life questionnaire; TSD-OC: Test SIO Disabilità Obesità Correlata; PAT-D: Pepper Assessment Tool for Disability; AE: Adverse event.

**Table S2**: Summary of Treatment-Emergent Treatment Related Adverse Events by System Organ Class and Preferred Term in the Safety Population. N = group number. [a] If a subject experienced more than one event within the same system organ class and preferred term, only one occurrence was included at each level of system organ class or preferred term. Totals for the number of subjects at system organ class level were not necessarily the sum of those at the preferred term levels since a subject could report two or more different adverse events within the higher-level category. [b] The total number of events of the type specified. Subjects could be represented more than once. For 'Any Treatment Related TEAE', it represents the total number of treatment related TEAEs. TEAE was defined as any event that starts on or after the first dose date of study drug up to the last dose date + 6 weeks (date of first randomized study medication intake ≤AE onset date ≤last dose date + 6 weeks). Adverse event was defined as related if causality was either definitely related, probably related or potentially related. Percentages (%) were based on number of subjects in the Safety Population.
