# Supplementary figures and images for: BIO101 in Sarcopenic Seniors at Risk of Mobility Disability: Results of a Double‐Blind Randomised Interventional Phase 2b Trial
Source: J Cachexia Sarcopenia Muscle. 2025 Mar 3;16(2):e13750. doi: 10.1002/jcsm.13750 (PMC11873539; doi:10.1002/jcsm.13750)

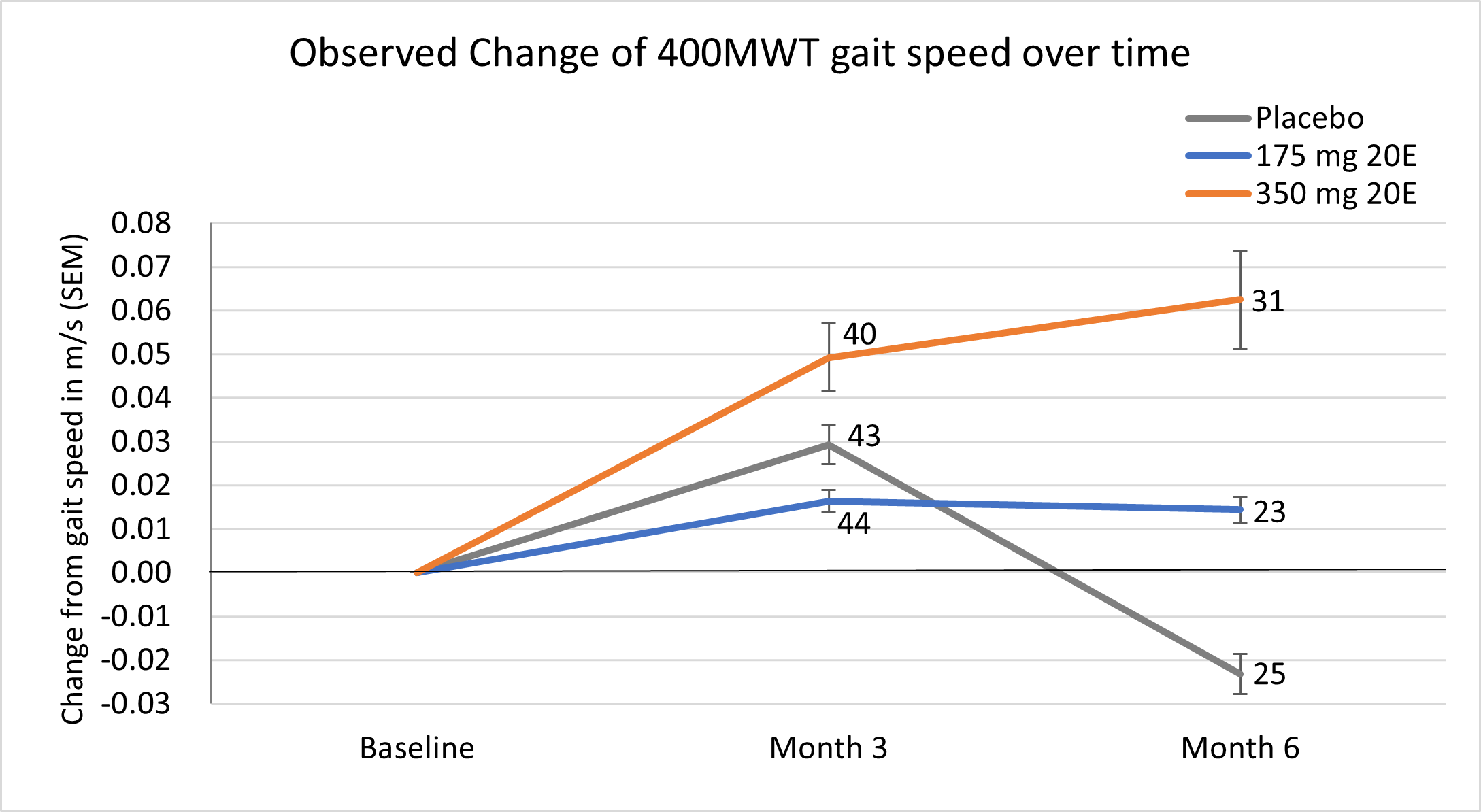

Supplement: Supplementary file 2 — Figure S1 Observed change from baseline at Month 6 of the 400MWT gait speed in meter per second (SEM) of the FAS population. Numbers indicate the number of observations at each timepoint and treatment arm. [file JCSM-16-e13750-s002.tif]

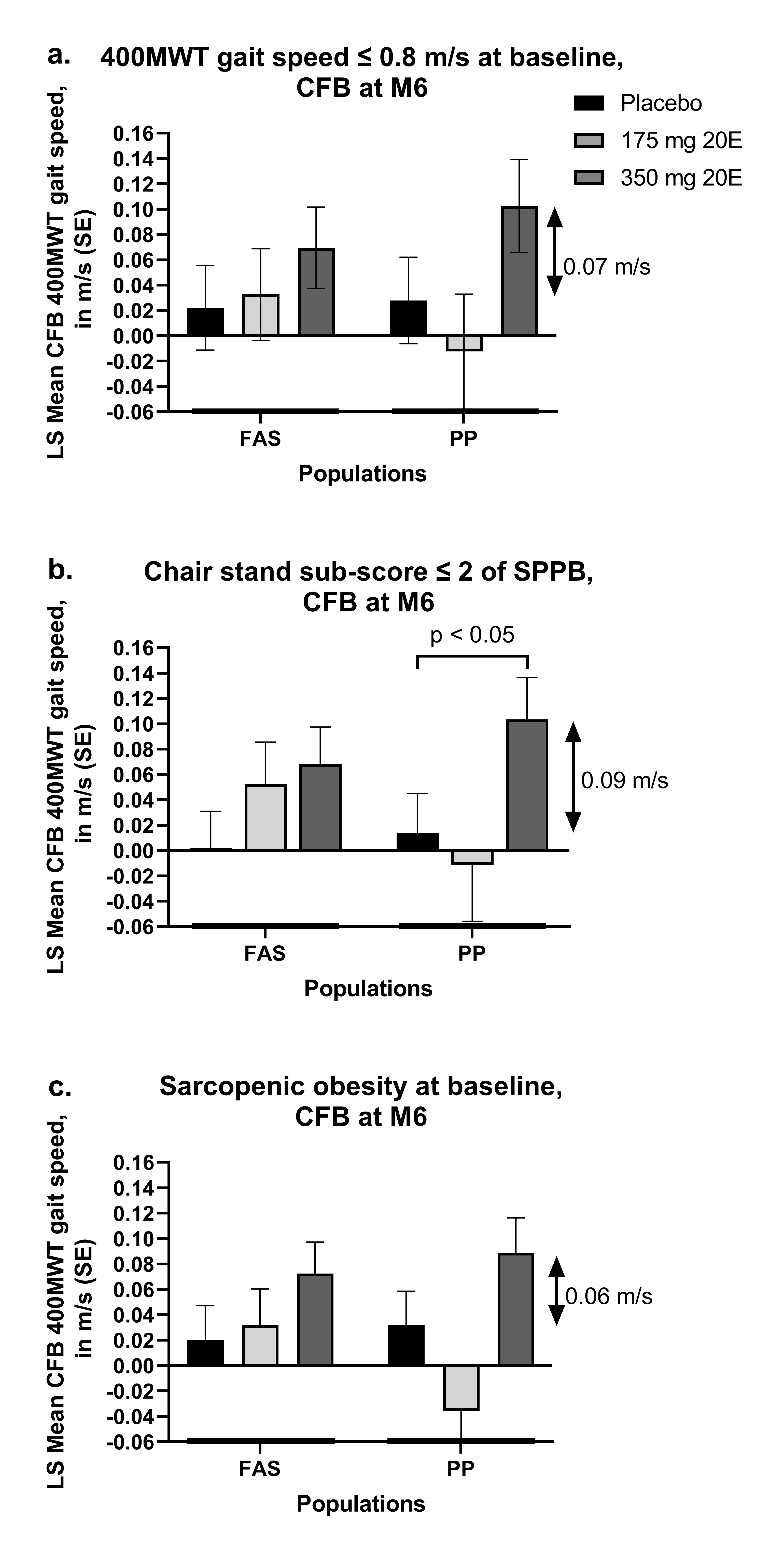

Supplement: Supplementary file 3 — Figure S2: Change from baseline in 400MWT gait speed (SE) at Month 6 in predefined subgroups: 400MWT gait speed ≤ 0.8 m/s at baseline (a), chair stand sub‐score ≤ 2 of SPPB (b) and sarcopenic obesity (c). Data are presented in FAS and PP populations. [file JCSM-16-e13750-s004.tif]
