# Supplementary material for: BIO101 in Sarcopenic Seniors at Risk of Mobility Disability: Results of a Double‐Blind Randomised Interventional Phase 2b Trial
Source: J Cachexia Sarcopenia Muscle. 2025 Mar 3;16(2):e13750. doi: 10.1002/jcsm.13750 (PMC11873539; doi:10.1002/jcsm.13750)
Supplement: Supplementary file 4 — Table S1: Schedule of activities. 400MWT, 400‐m walking test; AE, adverse event; CIRS, Cumulative Illness Rating Scale; DXA, dual‐energy X‐ray absorptiometry; EoS, end of study; ICF, Informed Consent Form; SarQoL: Sarcopenia Quality of Life questionnaire; SPPB, Short Physical Performance Battery; SF‐MNA, Short Form‐Mini Nutritional Assessment; SF36, Short Form 36; TSD‐OC, Test SIO Disabilità Obesità Correlata; PAT‐D, Pepper Assessment Tool for Disability. [file JCSM-16-e13750-s005.docx]

|  | **Screening**  **-8 -0 wks** | **Randomization& Baseline Visit** | **M1**  **visit** | **M3**  **visit** | **M5**  **phone call** | **M6**  **(EoS) visit^1^** | **M7.5**  **phone call^1^** | **M39**  **(EoS) visit^2^** | **M40.5**  **Post EoS Phone Call^2^** |
| --- | --- | --- | --- | --- | --- | --- | --- | --- | --- |
| ICF, demographics, Medical History | X |  |  |  |  |  |  |  |  |
| Concomitant medication | X |  | X | X |  | X^1^ |  | X^2^ |  |
| General safety questions |  |  | X |  | X |  | X^1^ | X^2^ | X^2^ |
| Safety measurements |  | X | X | X |  | X^1^ |  | X^2^ |  |
| Safety lab (haematology, biochemistry, urinalysis) | X |  | X | X |  | X^1^ |  | X^2^ |  |
| SPPB | X |  |  |  |  | X^1^ |  | X^2^ |  |
| DXA, Gallbladder ultrasound | X |  |  |  |  | X^1^ |  | X^2^ |  |
| Plasma and urine collection for biomarkers | X |  |  |  |  | X^1^ |  | X^2^ |  |
| 400MWT | X |  |  | X |  | X^1^ |  | X^2^ |  |
| Inclusion/  Exclusion criteria | X |  |  |  |  |  |  |  |  |
| **Randomization** |  | **X** |  |  |  |  |  |  |  |
| CIRS, SF-MNA |  | X |  |  |  |  |  |  |  |
| 6MWD, Grip strength |  | X |  |  |  | X^1^ |  | X^2^ |  |
| Stair Climb Power Test |  | X |  |  |  | X^1^ |  | X^2^ |  |
| Knee extension (optional) |  | X |  |  |  | X^1^ |  | X^2^ |  |
| SF-36, SarQoL, TSD-OC |  | X |  | X |  | X |  | X^2^ |  |
| PAT-D |  | X |  |  |  | X |  | X^2^ |  |
| Diabetes/prediabetes questions |  |  |  |  |  |  | X^1^ |  | X^2^ |
| AEs review and evaluation |  | X | X | X | X | X | X | X^2^ | X^2^ |

^1^ out of COVID-19 pandemic period (before March 2020 or after containment locally lifted)

^2^ during COVID-19 pandemic
