# Supplementary material for: BIO101 in Sarcopenic Seniors at Risk of Mobility Disability: Results of a Double‐Blind Randomised Interventional Phase 2b Trial
Source: J Cachexia Sarcopenia Muscle. 2025 Mar 3;16(2):e13750. doi: 10.1002/jcsm.13750 (PMC11873539; doi:10.1002/jcsm.13750)
Supplement: Supplementary file 5 — Table S2: Summary of treatment‐emergent treatment related adverse events by system organ class and preferred term in the safety population. N = group number. (a) If a subject experienced more than one event within the same system organ class and preferred term, only one occurrence was included at each level of system organ class or preferred term. Totals for the number of subjects at system organ class level were not necessarily the sum of those at the preferred term levels because a subject could report two or more different adverse events within the higher‐level category. (b) The total number of events of the type specified. Subjects could be represented more than once. For ‘any treatment‐related TEAE’, it represents the total number of treatment‐related TEAEs. TEAE was defined as any event that starts on or after the first dose date of study drug up to the last dose date + 6 weeks (date of first randomised study medication intake ≤ AE onset date ≤ last dose date + 6 weeks). Adverse event was defined as related if causality was either definitely related, probably related or potentially related. Percentages (%) were based on number of subjects in the safety population. [file JCSM-16-e13750-s003.docx]

| **System Organ Class**  **Preferred term** | **Placebo**  **(N=81)** | | **175mg 20E**  **(N=75)** | | **350mg 20E**  **(N=74)** | | **Overall**  **(N=230)** | |
| --- | --- | --- | --- | --- | --- | --- | --- | --- |
| **Subjects with AEs[a]/ Number of AEs[b]/** | **Subjects [a]** | **AEs [b]** | **Subjects [a]** | **AEs [b]** | **Subjects [a]** | **AEs [b]** | **Subjects [a]** | **AEs [b]** |
| Any related TEAE | 13 (16.0) | 24 | 10 (13.3) | 15 | 10 (13.5) | 16 | 33 (14.3) | 55 |
| Blood and lymphatic system disorders | 1 (1.2) | 1 | 1 (1.3) | 1 | 1 (1.4) | 2 | 3 (1.3) | 4 |
| Anaemia | 1 (1.2) | 1 | 0 | 0 | 1 (1.4) | 1 | 2 (0.9) | 2 |
| International normalized ratio increased | 0 | 0 | 1 (1.3) | 1 | 0 | 0 | 1 (0.4) | 1 |
| Thrombocytopenia | 0 | 0 | 0 | 0 | 1 (1.4) | 1 | 1 (0.4) | 1 |
| Endocrine disorders | 0 | 0 | 1 (1.3) | 1 | 0 | 0 | 1 (0.4) | 1 |
| Hypoglycaemia | 0 | 0 | 1 (1.3) | 1 | 0 | 0 | 1 (0.4) | 1 |
| Gastrointestinal disorders | 3 (3.7) | 4 | 4 (5.3) | 5 | 5 (6.8) | 7 | 12 (5.2) | 16 |
| Abdominal bloating | 1 (1.2) | 1 | 0 | 0 | 0 | 0 | 1 (0.4) | 1 |
| Abdominal discomfort | 0 | 0 | 1 (1.3) | 1 | 0 | 0 | 1 (0.4) | 1 |
| Acid reflux (oesophageal) | 1 (1.2) | 1 | 0 | 0 | 0 | 0 | 1 (0.4) | 1 |
| Alternation between constipation and diarrhoea | 1 (1.2) | 1 | 0 | 0 | 0 | 0 | 1 (0.4) | 1 |
| Bloated feeling | 0 | 0 | 0 | 0 | 1 (1.4) | 1 | 1 (0.4) | 1 |
| Constipation | 1 (1.2) | 1 | 0 | 0 | 1 (1.4) | 1 | 2 (0.9) | 2 |
| Diarrhoea | 0 | 0 | 1 (1.3) | 1 | 1 (1.4) | 2 | 2 (0.9) | 3 |
| Diarrhoea recurrent | 0 | 0 | 1 (1.3) | 1 | 0 | 0 | 1 (0.4) | 1 |
| Flatulence | 0 | 0 | 1 (1.3) | 1 | 0 | 0 | 1 (0.4) | 1 |
| Frequent bowel movements | 0 | 0 | 0 | 0 | 1 (1.4) | 1 | 1 (0.4) | 1 |
| Nausea | 0 | 0 | 1 (1.3) | 1 | 1 (1.4) | 1 | 2 (0.9) | 2 |
| Stomach pain | 0 | 0 | 0 | 0 | 1 (1.4) | 1 | 1 (0.4) | 1 |
| Hepatobiliary disorders | 2 (2.5) | 3 | 0 | 0 | 1 (1.4) | 1 | 3 (1.3) | 4 |
| Cholelithiasis | 0 | 0 | 0 | 0 | 1 (1.4) | 1 | 1 (0.4) | 1 |
| Gallbladder polyp | 1 (1.2) | 1 | 0 | 0 | 0 | 0 | 1 (0.4) | 1 |
| Impaired liver function | 1 (1.2) | 2 | 0 | 0 | 0 | 0 | 1 (0.4) | 2 |
| Investigations | 1 (1.2) | 1 | 1 (1.3) | 1 | 0 | 0 | 2 (0.9) | 2 |
| GGT increased | 0 | 0 | 1 (1.3) | 1 | 0 | 0 | 1 (0.4) | 1 |
| Lipase increased | 1 (1.2) | 1 | 0 | 0 | 0 | 0 | 1 (0.4) | 1 |
| Metabolism and nutrition disorders | 2 (2.5) | 3 | 1 (1.3) | 1 | 0 | 0 | 3 (1.3) | 4 |
| Decreased appetite | 1 (1.2) | 1 | 0 | 0 | 0 | 0 | 1 (0.4) | 1 |
| Hyperkalaemia | 1 (1.2) | 2 | 0 | 0 | 0 | 0 | 1 (0.4) | 2 |
| Hyperlipasaemia | 0 | 0 | 1 (1.3) | 1 | 0 | 0 | 1 (0.4) | 1 |
| Musculoskeletal and connective tissue disorders | 2 (2.5) | 3 | 4 (5.3) | 6 | 2 (2.7) | 2 | 8 (3.5) | 11 |
| Arthralgia | 0 | 0 | 1 (1.3) | 1 | 0 | 0 | 1 (0.4) | 1 |
| Arthropathy NOS | 0 | 0 | 0 | 0 | 1 (1.4) | 1 | 1 (0.4) | 1 |
| Leg cramps | 0 | 0 | 1 (1.3) | 1 | 0 | 0 | 1 (0.4) | 1 |
| Muscle soreness | 0 | 0 | 1 (1.3) | 1 | 0 | 0 | 1 (0.4) | 1 |
| Muscle weakness | 1 (1.2) | 1 | 0 | 0 | 0 | 0 | 1 (0.4) | 1 |
| Myalgia | 1 (1.2) | 2 | 1 (1.3) | 1 | 0 | 0 | 2 (0.9) | 3 |
| Myalgia of lower extremities | 0 | 0 | 2 (2.7) | 2 | 0 | 0 | 2 (0.9) | 2 |
| Osteoarthritis | 0 | 0 | 0 | 0 | 1 (1.4) | 1 | 1 (0.4) | 1 |
| Nervous system disorders | 1 (1.2) | 2 | 0 | 0 | 1 (1.4) | 3 | 2 (0.9) | 5 |
| Dizziness | 0 | 0 | 0 | 0 | 1 (1.4) | 1 | 1 (0.4) | 1 |
| Headache | 0 | 0 | 0 | 0 | 1 (1.4) | 1 | 1 (0.4) | 1 |
| Headache NOS | 1 (1.2) | 1 | 0 | 0 | 0 | 0 | 1 (0.4) | 1 |
| Insomnia | 0 | 0 | 0 | 0 | 1 (1.4) | 1 | 1 (0.4) | 1 |
| Light-headedness | 1 (1.2) | 1 | 0 | 0 | 0 | 0 | 1 (0.4) | 1 |
| Renal and urinary disorders | 1 (1.2) | 2 | 0 | 0 | 0 | 0 | 1 (0.4) | 2 |
| Urolithiasis | 1 (1.2) | 2 | 0 | 0 | 0 | 0 | 1 (0.4) | 2 |
| Respiratory, thoracic and mediastinal disorders | 0 | 0 | 0 | 0 | 1 (1.4) | 1 | 1 (0.4) | 1 |
| Coughing | 0 | 0 | 0 | 0 | 1 (1.4) | 1 | 1 (0.4) | 1 |
| Skin and subcutaneous tissue disorders | 2 (2.5) | 3 | 0 | 0 | 0 | 0 | 2 (0.9) | 3 |
| Dry skin | 1 (1.2) | 1 | 0 | 0 | 0 | 0 | 1 (0.4) | 1 |
| Itching | 1 (1.2) | 1 | 0 | 0 | 0 | 0 | 1 (0.4) | 1 |
| Itchy skin | 1 (1.2) | 1 | 0 | 0 | 0 | 0 | 1 (0.4) | 1 |
| Social circumstances | 1 (1.2) | 1 | 0 | 0 | 0 | 0 | 1 (0.4) | 1 |
| Fatigue | 1 (1.2) | 1 | 0 | 0 | 0 | 0 | 1 (0.4) | 1 |
| Vascular disorders | 1 (1.2) | 1 | 0 | 0 | 0 | 0 | 1 (0.4) | 1 |
| Hypotension orthostatic | 1 (1.2) | 1 | 0 | 0 | 0 | 0 | 1 (0.4) | 1 |
